# Isolation of a putative sulfur comproportionating microorganism

**DOI:** 10.1038/s41598-025-01009-y

**Published:** 2025-05-23

**Authors:** Heidi S. Aronson, Douglas E. LaRowe, Jennifer L. Macalady, Jan P. Amend

**Affiliations:** 1 Department of Biological Sciences, University of Southern California, Los Angeles, CA USA; 2 Department of Plant and Microbial Biology, University of California, Berkeley, CA USA; 3 Department of Earth Sciences, University of Southern California, Los Angeles, CA USA; 4 Department of Earth Sciences, Southern Methodist University, University Park, TX USA; 5 Department of Geosciences, Pennsylvania State University, University Park, PA USA

**Keywords:** Sulfur, Microbial catabolism, Acidophiles, Comproportionation, Novel metabolism, Biogeochemistry, Element cycles, Environmental microbiology

## Abstract

Sulfur comproportionation, the coupled oxidation of sulfide and reduction of sulfate to elemental sulfur, is a heretofore undiscovered microbial catabolism that has been predicted based on thermodynamic calculations. Here, we report the isolation of an *Acidithiobacillus thiooxidans* strain that grew to cell densities of > 10^7^ cells mL^−1^ in autotrophic sulfur comproportionation medium. It was isolated from extremely low pH snottite biofilms in the sulfidic karst at Frasassi, Italy. The organism’s genome sequence revealed the presence of numerous genes involved in sulfur transformations that could be linked in a sulfur comproportionation pathway. Future investigations of this novel link in the biogeochemical sulfur cycle could include monitoring of the concentrations of sulfate, sulfide, and elemental sulfur during growth to learn whether they are consistent with stoichiometric ratios expected for sulfur comproportionation. In addition, stable and radioisotope incubations coupled with NanoSIMS, scintillation counting, and isotope ratio mass spectrometry should reveal that sulfate and sulfide are coupled in a single catabolic reaction to produce elemental sulfur.

**Supplementary Information:**

The online version contains supplementary material available at 10.1038/s41598-025-01009-y.

## Introduction

The prediction of and search for novel microbial catabolisms can be streamlined by using thermodynamics to identify exergonic (i.e., energy-yielding) redox reactions mediated by microorganisms. This strategy has been successfully used to predict several previously overlooked microbial catabolic reactions, including anaerobic oxidation of ammonium, anaerobic oxidation of methane, and complete ammonia oxidation^1–3^. Sulfur comproportionation, or the reaction of sulfide and sulfate to form elemental sulfur (S^0^),

1 $$\:{\text{3 H}}_{\text{2}}\text{S}\text{}\text{+}\:{\text{SO}}_{\text{4}}^{\text{2-}}\text{+}\text{}\text{2}{\text{H}}^{\text{+}}\rightleftharpoons\:{\text{4 S}}^{\text{0}}\text{}\text{+}\:{\text{4 H}}_{\text{2}}\text{O}$$

was recently proposed as a novel microbial catabolic reaction on the basis of Gibbs energy calculations^4^. This reaction is exergonic in low-temperature, acidic environments with high sulfide and sulfate concentrations and could supply energy for microbial growth in such environments. Ideal conditions for sulfur comproportionation can be found within biofilms in the Frasassi cave system (Italy).

The caves at Frasassi are actively forming through a process called sulfuric acid speleogenesis (SAS), where sulfide in the groundwater degasses into the cave air and is oxidized to form sulfuric acid^5,6^. Sulfide-oxidizing microorganisms can accelerate the production of sulfuric acid and consequently contribute to speleogenesis^7–9^. Gypsum (CaSO_4_∙2H_2_O) formed through SAS is deposited in crusts on the limestone walls, and microbial biofilms cover the walls at the gypsum-air interface^10–12^. These biofilms, termed “snottites”, hang from the surface of the gypsum and are also associated with elemental sulfur^11^. Snottites that are slightly removed from the buffering on the limestone and gypsum are extremely acidic (pH 0–1). These biofilms exist in a cool environment (13.4ºC) and are exposed to sulfide in the cave air, and sulfate and extreme acidity from sulfuric acid, making them the ideal habitat for sulfur comproportionators. Here, we report the isolation of a strain of *Acidithiobacillus thiooxidans* from Frasassi snottites which is putatively capable of growth by sulfur comproportionation.

## Methods

### Sample collection

Approximately 0.5 cm^3^ of snottite biofilm was collected from Ramo Sulfureo in the Frasassi cave system in July 2019 as inoculum for a custom growth medium targeted for the enrichment of autotrophic sulfur comproportionators. Snottite pH was measured in the field with pH paper (range 0–2.5). H_2_S_(g)_ concentrations were measured above the stream using an ENMET RECON/4a portable gas detector. A 100 mL serum bottle containing 50 mL sterile, anoxic comproportionation cultivation medium (described below) and sealed with a blue butyl stopper was brought into the cave. Immediately before snottites were removed from the cave wall using sterile forceps, a sterile syringe and needled were used to transfer 1 mL of sterile medium into a microcentrifuge tube. The snottite sample was placed into the tube and the same syringe was immediately used to transfer the snottite-medium slurry into the serum bottle.

### Cultivation

Custom cultivation medium for sulfur comproportionation was prepared under 80% N_2_−20% CO_2_. The basal medium contained the following (per liter of 50 mM sulfuric acid): 2.42 g Na_2_SO_4_, 0.50 g MgSO_4_·7H_2_O, 0.025 g CaCl_2_·2H_2_O, 0.1 g KCl, 0.13 g (NH_4_)_2_SO_4_, 0.14 g Na_2_HPO_4_, 1 mL trace element solution, and 0.5 mL of 0.2% resazurin. The pH was adjusted to 1 with concentrated sulfuric acid and the medium was dispensed into serum bottles. Bottles were stoppered and crimped, and a slight overpressure was added to the headspace with 80% N_2_−20% CO_2_. After autoclaving, the medium was amended with 1 mL of 1000X vitamin solution, sodium bicarbonate at a final concentration of 10 mM, and Na_2_S·9H_2_O at a final concentration of 2 mM. The 1000X trace element solution contained the following (per liter of water): 1.7 mL 20 mM HCl, 2.10 g FeSO_4_·7H_2_O, 0.03 g H_3_BO_3_, 0.10 g MnCl_2_·4H_2_O, 0.19 g CoCl_2_·6H_2_O, 0.024 g NiCl_2_·6H_2_O, 0.002 g CuCl_2_·6H_2_O, 0.144 g ZnSO_4_·7H_2_O, 0.036 g Na_2_MoO_4_·2H_2_O, 0.0326 g VOSO_4_·H_2_O, 0.025 Na_2_WO_4_·2H_2_O, 0.006 g Na_2_SeO_3_·5H_2_O. The 1000X vitamin solution contained the following (per liter 10 mM MOPS, pH 7.2): 0.10 g riboflavin, 0.03 g biotin, 0.10 g thiamine HCl, 0.10 g L-ascorbic acid, 0.10 g D-calcium pantothenate, 0.10 g folic acid, 0.10 g nicotinic acid, 0.10 g 4-aminobenzoic acid, 0.10 g pyridoxine HCl, 0.10 g lipoic acid, 0.10 g thiamine pyrophosphate, 0.01 g cyanocobalamin.

Incubation conditions were selected that yield the same amount of energy for sulfur comproportionation as the sample site. Inoculated bottles were incubated at 15ºC until return to the laboratory at the University of Southern California. The culture was incubated at 15ºC and transferred to fresh media once per month for 4 months, then purified by dilution to extinction. Purity of the resulting isolate was confirmed by Sanger sequencing of the 16 S rRNA gene.

### Cell counting

500 µL culture samples were fixed with glutaraldehyde (2.5% final concentration), incubated at room temperature for 1 h, neutralized with 5 M NaOH, and stained with 10X SYBR Green I. SYBR Green I was diluted from a 10,000X stock in a pH 8.0 Tris-EDTA solution. Neutralization of the fixed culture with NaOH was performed because SYBR Green I is pH sensitive^13^. Samples were filtered through 25 mm black polycarbonate filters and visualized under a fluorescence microscope. 50 fields of view were counted across each filter. Fixed samples were diluted in sterile milliQ water so that each field of view contained approximately 50 cells.

### Thermodynamic calculations

The Gibbs energy yields (∆*G*_*r*_) for sulfur comproportionation and sulfate reduction coupled to ammonium oxidation were calculated with

2 $$\:\varDelta\:{G}_{r}=\varDelta\:{G}_{r}^{0}+RT\text{l}\text{n}{Q}_{r}$$

where $\:\varDelta\:{G}_{r}^{0}$ is the standard state Gibbs energy, *R* is the universal gas constant, *T* is the temperature in Kelvin, and *Q*_*r*_ refers to the reaction quotient. Values of $\:\varDelta\:{G}_{r}^{0}$ were calculated at in situ or media temperatures and 1 bar with the revised Helgeson-Kirkham-Flowers (HKF) equations of state^14–16^ using the “subcrt” command from the R software package CHNOSZ v1.4.1^17^. Thermodynamic data in CHNOSZ are derived from the OrganoBioGeoTherm database, which come from a number of sources, as documented in (https://chnosz.net/download/refs.html). Values of *Q*_*r*_ were calculated with

3 $$\:{Q}_{r}={\Pi\:}{a}_{i}^{{\nu\:}_{i,r}}$$

where *a*_*i*_ represents the activity of the *i*^th^ species raised to its stochiometric reaction coefficient ν_*i, r*_, in the *r*^th^ reaction, which is positive for products and negative for reactants.

Activities were calculated with the relation

4 $$\:{a}_{i}={\gamma\:}_{i}\left(\frac{{C}_{i}}{{C}_{i}^{{\Theta\:}}}\right)$$

where *γ*_*i*_ and *C*_*i*_ are the activity coefficient and concentration of the *i*^th^ species, respectively. *C*_*i*_^Θ^ is the concentration of the *i*^th^ species under standard state conditions, which is equal to one molal referenced to infinite dilution. Activities for aqueous species were determined using the aqueous speciation package AqEquil v0.9.1^18^, which is based on EQ3/6 ^19^. The activities of S^0^ and water were taken to be unity (*a*_*i*_ = 1). Concentrations were sourced from the sulfur comproportionation medium recipe and from in situ geochemical concentrations. H_2_S_(g)_ concentrations in the cave air ranged from 15 to 33 ppmv. Sulfate concentration in the snottite was inferred from the pH (0–1).

The Gibbs energy for sulfate reduction coupled to ammonium oxidation was calculated under medium conditions at 15ºC for six reactions with different S and N products to demonstrate that sulfate could not be reduced by any other reductants in the system (Supplemental Table 1).

## DNA extraction and bioinformatics

Samples for DNA extraction were collected by filtering 2 mL enrichment medium through a sterile 25 mm, 0.1 μm Supor filter. DNA for 16 S rRNA gene sequencing was extracted from filters using the Qiagen PowerBiofilm DNA extraction kit following manufacturer instructions. Full-length 16 S rRNA genes were amplified from isolate cultures using the 27 F/1492R primer pair. Sanger sequencing was performed by GeneWiz (La Jolla, CA). DNA for whole genome sequencing was extracted from filters using a combination of proteinase K digestion, phenol-chloroform-isoamyl alcohol extraction, and ethanol precipitation (https://www.protocols.io/view/modified-phenol-chloroform-genomic-dna-extraction-e6nvwkjzwvmk/v2). Whole genome sequencing was performed at the Caltech Genetics and Genomics Laboratory. Oxford Nanopore sequencing libraries were constructed using the PCR Barcoding Kit (SQK-PBK004) and were sequenced using MinION flowcells (FLO-MIN106). Basecalling was performed with ONT Guppy v3.4.5. Illumina libraries were constructed using the NEBNext Ultra kit. Single end, 100 basepair reads were sequenced using HiSeq2500.

Nanopore long reads were assembled using Canu v.2.1.1^20^. Reads were polished with Racon v1.4.20^21^ and Medaka v1.1.1 (https://github.com/nanoporetech/medaka). Reads with Q scores below 7 were filtered and barcodes and adapters were trimmed. The resulting assembly was mapped using BamM (http://ecogenomics.github.io/BamM/) with 150 bp Illumina paired-end reads. Mapped reads were used for error correction using Pilon v1.2.2^22^. Genome quality was determined using CheckM2 v1.0.1^23^. Closely related strains were identified using GToTree^24^. The command “gtt-get-accessions-from-GTDB” was used to collect representative genomes from the genus *Acidithiobacillus* that were present in the Genome Taxonomy Database as of March 5, 2025^25^, and the command “GToTree” was used to construct a de novo phylogenomic tree of 172 Gammaproteobacterial single copy genes with the program IQ-TREE2^26^ (Supp. Figure 1). Average nucleotide identity (ANI) and average amino acid identity (AAI) were calculated for strain RS19-104 and *Acidithiobacillus thiooxidans* ATCC 19377^T^ using the IMG ANIcalculator^27^ and EzAAI^28^. Metabolic functional traits were predicted using METABOLIC v4.0^29^, MetaSanity v1.3.0^30^, and eggNOG-mapper v2^31^ (Supplemental Tables 3–9).

## Results and discussion

**Fig. 1:**
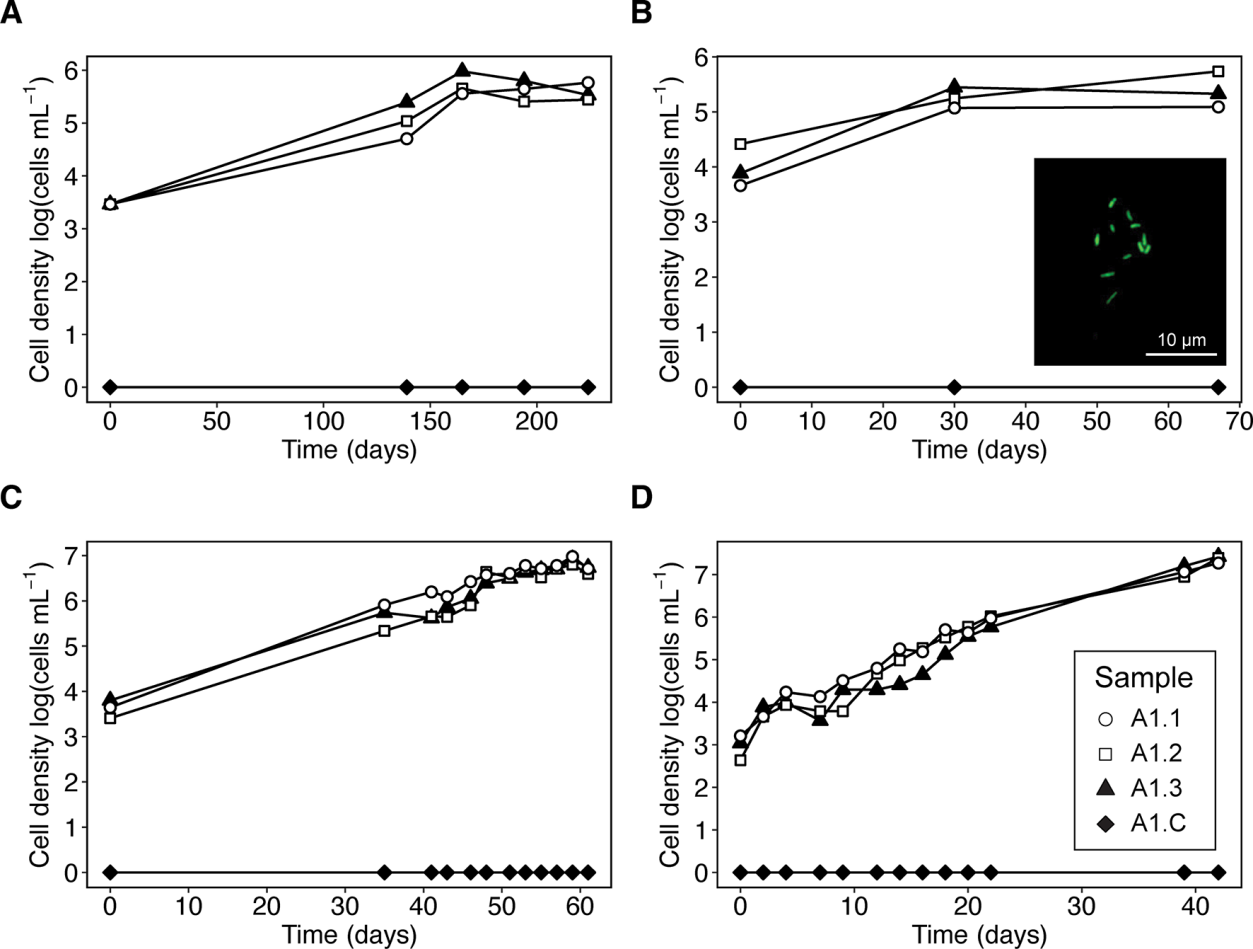
Cell densities in log (cells ml^−1^) of strain RS19-104 as a function of time. Cell densities were measured by direct cell counts via fluorescence microscopy. Each panel represents a separate transfer (A, transfer 1; B, transfer 2; C, transfer 3; D, transfer 4) of three inoculated bottles (samples A1.1, A1.2, A1.3) with an uninoculated negative control bottle (A1.C, black diamonds). The inset image in panel B shows cells of strain RS19-104 visualized under fluorescence microscopy. Scale bar represents 10 μm.

Sulfur comproportionation yields an in-situ Gibbs energy of 52 kJ mol^−1^. A bacterial strain enriched from a Frasassi cave snottite biofilm on custom autotrophic comproportionation medium was isolated by dilution to extinction transfers after four months of incubation at 15ºC. The resulting isolate was identified as *Acidithiobacillus thiooxidans* with 99.86% sequence similarity to the type strain in the full-length 16 S rRNA gene and was designated strain RS19-104. Cultures of strain RS19-104 (*n* = 12) showed increases in cell density of 2–3 orders of magnitude over the course of 1–2 months, with the highest cell densities reaching > 10^7^ cells mL^−1^ (Fig. 1). Cells of strain RS19-104 were rod-shaped and 1–2 μm in length (Fig. 1 inset).

*A. thiooxidans*, an autotrophic, sulfur-oxidizing acidophilic bacterium, is abundant in Frasassi snottite biofilms, making up over 70% of the microbial community there^11^, and is thought to be the dominant primary producer in the snottites. Because *A. thiooxidans* can grow via aerobic oxidation of reduced sulfur compounds, possible oxygen contamination of the growth medium was considered. However, several lines of evidence suggest that strain RS19-104 was not growing as an aerobic sulfide oxidizer. Anaerobic cultivation techniques were strictly applied with RS19-104 cultures, which included using blue butyl stoppers, adding a slight N_2_/CO_2_ overpressure in the headspace to prevent gas from leaking into the serum bottles, flushing needles with N_2_, working in an anaerobic chamber whenever possible, using resazurin as a redox indicator, and adding 2 mM sulfide as a reductant and electron donor. We therefore conclude that oxygen leakage cannot explain the growth of *A. thiooxidans* RS19-104 that we observed.

The draft genome of strain RS19-104 was 100% complete with 2.07% contamination and an average nucleotide identity and amino acid identity with *A. thiooxidans* ATCC 19377^T^ of 98.62% and 98.51%, respectively. The genome contained a complete pathway for carbon fixation via the Calvin-Benson-Bassham cycle, with RuBisCO, phosphoglycerate kinase, glyceraldehyde-3-phosphate dehydrogenase, transketolase, ribose-5-phosphate isomerase, and phosphoribulokinase.

Numerous genes encoding enzymes related to sulfur cycling were identified in the genome (Supplemental Tables 3–9). There are three possibilities for what a sulfur comproportionation pathway might look like in a genome. Because many enzymes involved in sulfur cycling are reversible, the first possibility is that a comproportionation pathway could use a S^0^ disproportionation pathway run in the reverse direction. However, there are not any genes or pathways associated with disproportionation that have been identified^32,33^, thus making the search for such pathways difficult. However, under anaerobic conditions, *At. ferrooxidans* disproportionates S^0^ to form H_2_S and SO_4_^2−^. Because *At. ferrooxidans* can disproportionate S^0^ under acidic conditions, it could serve as an ideal model organism for testing growth under comproportionation conditions.

**Fig. 2:**
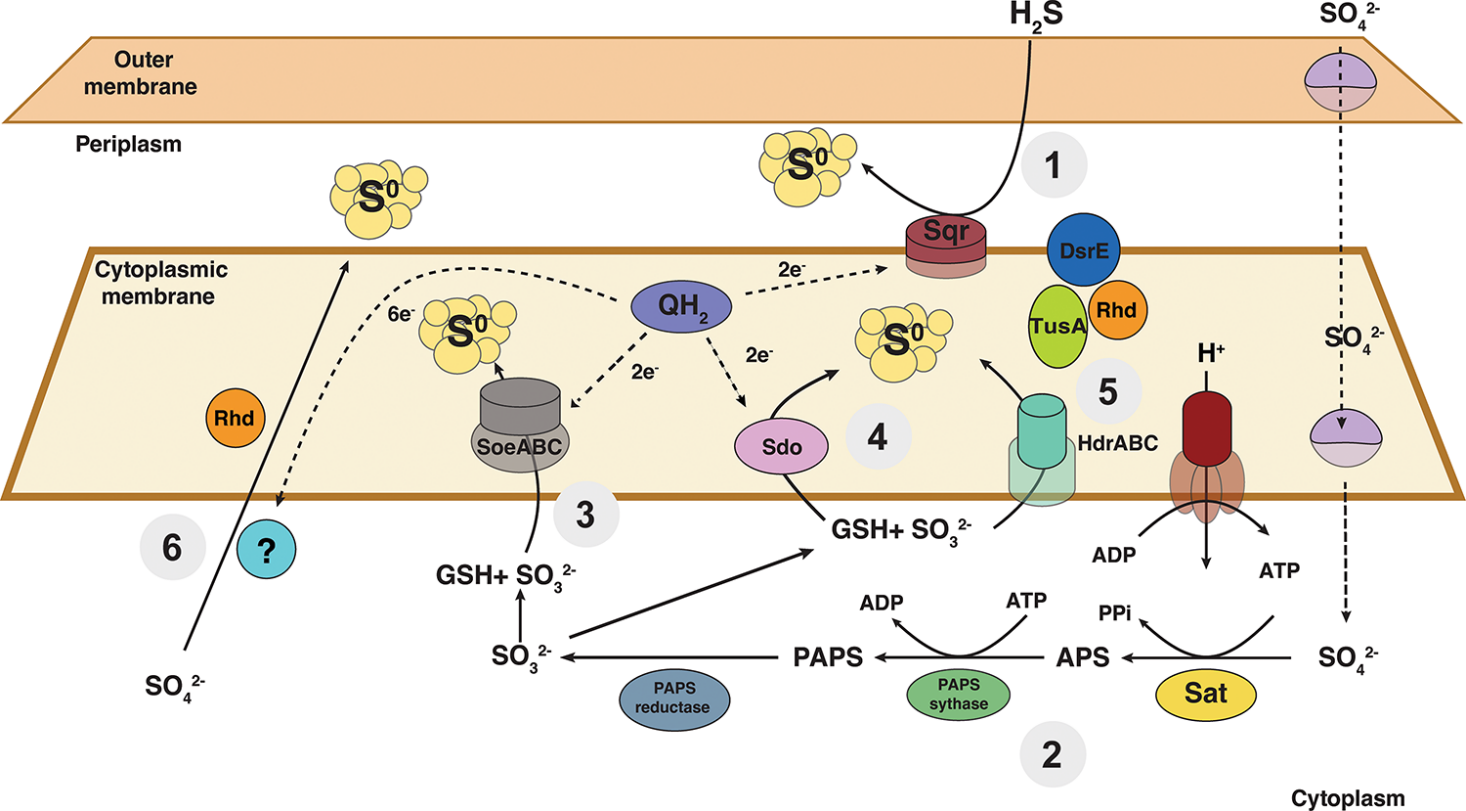
Proposed schematic of the comproportionation pathways of strain RS19-104 based on genes present in the whole genome sequence. Dashed lines indicate electron transfer reactions. Reaction 1: sulfide oxidation to S^0^ with Sqr. Reaction 2: sulfate reduction to sulfite with Sat, PAPS synthase, and PAPS reductase. Reaction 3: sulfite reduction to S^0^ with SoeABC. Reaction 4: sulfite reduction to S^0^ with *sdo*. Reaction 5: sulfite reduction to S^0^ with HdrABC and the sulfurtransferases DsrE, TusA, and Rhd. Reaction 6: direct reduction of sulfate to S^0^ with an unknown enzyme. The blue circle with a question mark indicates an unknown enzyme that could catalyze the direct reduction of sulfate to S^0^. Rhd may be involved in shuttling S^0^ to the periplasmic space. Gene names can be found in the main text. GSH represents glutathione.

The second possibility is that, because sulfur comproportionation is a novel microbial metabolism, it utilizes a set of completely novel genes and pathways. Like a reverse disproportionation pathway, it is difficult to identify unknown genes and pathways in a genome. The third possibility is that a sulfur comproportionating microorganism might use known sulfide oxidation and sulfate reduction pathways linked in previously unexpected ways. Because *A. thiooxidans* grows via aerobic sulfide oxidation using sulfide-quinone reductase (*sqr*), we postulated that Sqr might be used under comproportionation conditions to oxidize sulfide and donate electrons to the quinone pool (Fig. 2, reaction 1). However, it was unknown whether strain RS19-104 could use sulfate as an electron acceptor. Several species of *Acidithiobacillus* are facultatively capable of anaerobic respiration using Fe(III) or S^0^ as electron acceptors^34–40^, suggesting that anaerobic growth using sulfate as an electron acceptor is plausible. The presence of a complete dissimilatory sulfate reduction pathway (*sat*,* aprAB*,* dsrAB*) would indicate that strain RS19-104 uses sulfate for respiration. While *sat* was present in the genome, *aprAB* and *dsrAB* were absent. Instead, genes associated with the assimilatory sulfate reduction pathway, including *sat*, *cysC*, and *cysH* were present, suggesting that strain RS19-104 has the potential to produce sulfite from sulfate (Fig. 2, reaction 2). The assimilatory sulfate reduction pathway consumes 2 ATP to convert sulfate into sulfide destined to make the S-bearing amino acids cysteine and methionine. However, the genome of strain RS19-104 also contains the gene *cysM*, which encodes an enzyme used to assimilate sulfide directly from the environment. Because strain RS19-104 grows in a sulfide-rich environment, it is plausible that it would use sulfide for biosynthesis rather than invest energy into sulfate assimilation. We propose that strain RS19-104 could be using the assimilatory sulfate reduction pathway for comproportionation. Alternatively, a gene encoding SoeABC, which oxidizes sulfite to sulfate while donating electrons to the quinone pool, was also found in the genome^41,42^. If acting in reverse, SoeABC could catalyze the reduction of sulfate to form sulfite (Fig. 2, reaction 3).

Once sulfite is produced from sulfate reduction, there are several enzymes that, if acting in reverse, could catalyze the conversion of sulfite to S^0^. SoeABC was shown to operate in the reverse direction by reducing S^0^, thiosulfate, tetrathionate, and sulfite in vitro^42^. While the bidirectionality of SoeABC was not confirmed in vivo, it is possible that it could reduce sulfite to S^0^ in strain RS19-104 (Fig. 2, reaction 3). A gene encoding sulfur dioxygenase (*Sdo*) was present, which oxidizes S^0^ to sulfite in *A. thiooxidans*^43^ (Fig. 2, reaction 4). Several copies of the heterodisulfide reductase complex genes (*hdrABC*) were present in the genome of strain RS19-104. In lithotrophic sulfur oxidizers, HdrABC is thought to oxidize S^0^ to sulfite with the assistance of rhodanese, DsrE, and TusA-like sulfurtransferases^44^. The Hdr complex operon found in *Acidithiobacillus ferrooxidans* is also conserved in several acidophilic sulfur-oxidizing taxa^45,46^ as well as in strain RS19-104. Unlike HdrA in methanogenic archaea, HdrA in sulfur oxidizers is thought to be non-electron-bifurcating because it lacks central and carboxy-terminal ferredoxin-binding domains as well as an N terminal [4 Fe-4 S] cluster-binding domain^44^. These domains were also absent from HdrA in strain RS19-104. If it is unable to bind and accept electrons from ferredoxin, HdrA may accept electrons from the quinone pool to reduce sulfite to S^0^ (Fig. 2, reaction 5).

It is also possible that sulfate could be reduced directly to S^0^ under comproportionation conditions (Fig. 2, reaction 6). Although numerous oxidative processes produce S^0^ from sulfide or thiosulfate (e.g., sqr, sox pathway), canonical sulfate reduction proceeds through sulfite and then directly to sulfide without forming S^0^ as a product. Recent studies suggest that alternative reductive pathways may lead to the formation of S^0^. It was shown that phylogenetically diverse sulfate reducing microorganisms produce S^0^ in a pathway using some of the canonical dissimilatory sulfate reduction enzymes^47^. Supported by isotopic labeling, anaerobic methanotrophic (ANME) archaea have also been shown to perform direct reduction of sulfate to S^0^ under extremely reducing conditions^48^. Because sulfate reduction genes were absent in the genome, it was proposed that sulfate reduction by ANME archaea was catalyzed by a novel enzymatic pathway. Although this novel pathway has not yet been verified, a similar pathway could also be used by comproportionating microbes to produce S^0^ from sulfate (Fig. 2, reaction 6). Rhd-like sulfurtransferases are abundant in microorganisms grown under sulfur disproportionating conditions and are proposed to catalyze many reactions involving S^0^^49–51^. Under comproportionation conditions, Rhd may be used to shuttle S^0^ to the periplasmic space where it can accumulate. Additionally, it was recently shown that assimilatory sulfite reductases produce S^0^ from sulfite^52^. While this gene was not present in the genome of strain RS19-104, it is evidence to suggest that S^0^ can be a direct intermediate in sulfate reduction processes and could be used in a comproportionation mechanism.

In our proposed comproportionation pathway, sulfide is oxidized via Sqr to S^0^, donating two electrons to the quinone pool (Fig. 2). Sulfate is reduced either to sulfite by the assimilatory sulfate reduction pathway or to S^0^ by a novel metabolic pathway. The sulfite produced by sulfate reduction is reduced to S^0^ by reverse-acting Sdo, HdrABC, or SoeABC.

Although strain RS19-104 grew under comproportionation conditions for several transfers (Fig. 1) and we obtained enough biomass for whole genome sequencing, we were unable to maintain the culture growth for long periods. While the culture was ultimately lost, we developed and attempted an experimental framework that was ultimately unsuccessful due to insufficient biomass. We provide our recommendations and improvements to this experimental framework that can be applied to future searches for sulfur comproportionators in other natural and impacted environments with favorable conditions, including extreme acid mine drainage pit lakes, certain terrestrial volcanic springs and shallow marine vents, subglacial lakes, and acid-sulfate crater lakes (Supplementary Methods).

Future investigations of sulfur comproportionation should include monitoring of the concentrations of sulfate, sulfide, and elemental sulfur during growth to learn whether they are consistent with stoichiometric ratios expected for sulfur comproportionation (Rxn. 1). In addition, stable and radioisotope incubations coupled with NanoSIMS, scintillation counting, and isotope ratio mass spectrometry should reveal whether sulfate and sulfide are coupled in a single catabolic reaction to produce elemental sulfur. Multi-isotope incubations could be conducted to show that S atoms from sulfate and sulfide are both incorporated into S^0^ produced during cell growth (Supplementary Methods). Under aerobic sulfide oxidation conditions, *A. thiooxidans* accumulates S^0^ inclusions that can be observed by TEM^53^. If comproportionation produces similar intracellular S^0^, these inclusions should be visible with TEM and isotopic labels from sulfate and sulfide should be detected in intracellular inclusions via NanoSIMS. Additionally, because of the high background concentration of sulfate and sulfide in the comproportionation culture medium, radioisotope incubations may be advantageous for measuring reactant consumption rates.

Future searches for sulfur comproportionators could also use a genomics-guided approach to cultivation. Metagenome-assembled genomes from target environments that have both sulfide oxidation and dissimilatory sulfate reduction pathways could be targeted for cultivation with sulfur comproportionation media such as the one employed here. For example, genomes of the candidate lineage Acidulodesulfobacterales were reconstructed from an acid mine drainage system and contained both the dissimilatory sulfate reduction genes *dsrAB*,* dsrD*,* dsrL*, and *dsrEFH* and the sulfide oxidation genes *sqr*,* soxAB*, and *soxYZ*^54^. Because these pathways are present in a single genome, it is plausible that microorganisms exist which anaerobically oxidize sulfide with sulfate in a single comproportionation reaction.

## Electronic supplementary material

Below is the link to the electronic supplementary material.

Supplementary Material 1

Supplementary Material 2

Supplementary Material 3

## Data Availability

The 16S rRNA sequence and whole genome sequence are available in GenBank under accession numbers OR527823 and JAVKVP000000000.
