# Supplementary material for: Isolation of a putative sulfur comproportionating microorganism
Source: Sci Rep. 2025 May 23;15:17999. doi: 10.1038/s41598-025-01009-y (PMC12102312; doi:10.1038/s41598-025-01009-y)
Supplement: Supplementary file 1 — Supplementary Material 1 [file 41598_2025_1009_MOESM1_ESM.docx]

***Supplementary methods***

The following sections contain the analytical methodologies which we developed and attempted for this study. These experiments were inconclusive due to insufficient biomass, and we include recommendations for how these methods, which include chemical measurements, microscopy, and multi-isotope probing, could be improved for future sulfur comproportionation studies. The Gibbs energy yields for various ammonia-sulfate reactions are presented in Supplemental Table 1 to rule out the possibility of these catabolic reactions occurring in our cultures.

*Experimental setup:*

In addition to experimental inoculated conditions with sulfate and sulfide, the following control conditions should be included to demonstrate growth and sulfur comproportionation in the cultures. An uninoculated control condition and an inoculated, heat-killed control condition should be included to consider the possibility of abiotic sulfur comproportionation. Conditions without the electron donor (sulfide) or acceptor (sulfate) should be included to demonstrate that growth does not occur in the absence of either compound. The experimental and control conditions should be coupled with chemical measurements of sulfate, sulfide, and S^0^, described below.

*Chemical measurements*

Concentrations of sulfate, sulfide, and S^0^ were measured in comproportionation cultures using ion chromatography, the Cline assay (1), and high performance liquid chromatography, respectively. A method for extracting and measuring S^0^ is described in McGuire and Hamers 2000 (2). Samples were preserved and sulfur pools were separated as outlined below in the Radioisotope and Stable Isotope probing methodology.

*Electron microscopy*

Intracellular S^0^ inclusions may be visible via transmission electron microscopy. We were unable to obtain visible cell pellets even at cell densities of ≥10^7^ cells mL^-1^, so we recommend that large volumes (at least 25 mL) of culture should be grown and pelleted via centrifugation. Ultracentrifugation can be used to improve pelleting (3). Cell pellets should be rinsed with 0.1 M phosphate buffer, 0.1 M sodium cacodylate buffer, or 0.1 M HEPES buffer pH 7.2-7.4 to remove acidic medium, fixed with 4% paraformaldehyde and 0.1% glutaraldehyde, washed again three times with 0.1 M phosphate buffer, and stained with 1% osmium tetroxide for 1 hour at room temperature (4). The pellets should be dehydrated in a graded ethanol series and embedded in epoxy resin and ultrathin sectioned. Ultrathin sections should be stained with 2% uranyl acetate and lead citrate and then examined under transmission electron microscopy.

*Multi-isotope probing and nanoSIMS*

Multi-isotope incubations were performed using ${}_{\text{ }}^{\text{34}}{\text{S}_{\text{SO}_{\text{4}}^{\text{2-}}}}$ (90 atom% ^34^S, Sigma Aldrich) and ${}_{\text{ }}^{\text{33}}{\text{S}_{\text{H}_{\text{2}}\text{S}}}$. ${}_{\text{ }}^{\text{33}}{\text{S}_{\text{H}_{\text{2}}\text{S}}}$ was prepared by the reduction of ^33^S^0^ (99%, Sigma Aldrich). Fe^33^S was synthesized from ^33^S^0^ and carbonyl iron (Sigma Aldrich, ≥97% Fe basis). ^33^S^0^ and an excess of carbonyl iron were mixed in a Hungate tube sealed with a butyl rubber stopper and the headspace was flushed for 30 minutes with N_2_. The mixture was then ignited with an ethanol burner flame. The Fe^33^S was placed into a 150 mL serum bottle sealed with a butyl rubber stopper and the headspace was flushed for 30 minutes with N_2_, then placed on ice. 6.0 M HCl was sparged for 20 minutes with N_2_ on ice. To form ${}_{\text{ }}^{\text{33}}{\text{S}_{\text{H}_{\text{2}}\text{S}}}$, HCl was added with an N_2_-sparged syringe to the serum bottle containing Fe^33^S. The bottle was removed from the ice bath after 10 minutes and incubated at room temperature overnight. The following day, the bottle was placed in a sonicator bath to dissolve remaining Fe^33^S particles. Cold distillation of ${}_{\text{ }}^{\text{33}}{\text{S}_{\text{H}_{\text{2}}\text{S}}}$ was performed by flushing the bottle containing ${}_{\text{ }}^{\text{33}}{\text{S}_{\text{H}_{\text{2}}\text{S}}}$ with N_2_ for 10-15 minutes through 1.0 mM NaOH to trap NaH^33^S. The NaH^33^S was neutralized with HCl to form ${}_{\text{ }}^{\text{33}}{\text{S}_{\text{H}_{\text{2}}\text{S}}}$. Conversion to H_­2_S was verified and quantified with the Cline assay (1).

Modified comproportionation medium was prepared with 30 mM H_2_SO_4­_ (instead of 50 mM) without the addition of Na_2_SO_4_ and with only 0.5 mM (NH_4_)_2_SO_4_. 10 mL of medium was dispensed into Balch tubes under 80% N_2_-20% CO_2_, and the tubes were stoppered and autoclaved. After autoclaving, the medium was amended with sodium bicarbonate at a final concentration of 5 mM and Na_2_S x 9H_2_O at a final concentration of 1.6 mM. The following isotopically labeled compounds were added: ${}_{\text{ }}^{\text{34}}{\text{S}_{{\text{Na}_{\text{2}}\text{SO}}_{\text{4}}^{\text{2-}}}}$ (final concentration 20 mM; 40 at. %), ${}_{\text{ }}^{\text{33}}{\text{S}_{\text{H}_{\text{2}}\text{S}}}$ (final concentration 0.4 mM; 20 at. %), ${}_{\text{ }}^{\text{13}}{\text{C}_{\text{HCO}_{\text{3}}^{\text{-}}}}$ (final concentration 5 mM; 50 at. %), and ${}_{\text{ }}^{\text{15}}{\text{N}_{\text{NH}_{\text{4}}\text{Cl}}}$ (final concentration 0.5 mM; 50 at. %). The at. % added to incubations are calculated by isotope mass balance: ^n^F_final_ = [(^n^F_unlabeled_ x m_unlabeled_) + (^n^F_labeled_ x m_labeled_)]/m_final_, where mass (m) was the amount of Na_2_SO_4_, H_2_S, NH_4_Cl, or HCO_3_^-^ added to the mixture and at. % = 100 x ^n^F. The final medium was inoculated with sulfur comproportionation cultures. After 1 month of incubation at 15ºC, samples was fixed with glutaraldehyde (final concentration 2.5%), filtered onto a 0.1 µm, 13 mm Supor filter, dehydrated in an ethanol series, and coated with 20 nm gold. Carbon, nitrogen, and sulfur isotopic compositions were measured using a NanoSIMS 50L (CAMECA, Gennevilliers, France). Cells were analyzed using a ~4 pA primary Cs^+^ beam current. Seven masses were collected in parallel (^12^C, ^13^C, ^14^N, ^15^N, ^32^S, ^33^S, and ^34^S). We were unable to locate cells during NanoSIMS analysis. To concentrate the cells in a small area on the filter, the Supor filter disk should be placed on a vacuum filtration tower and cell culture should be added with a micropipette.

*Radioisotope and stable isotope probing*

Sulfur comproportionation cultures were used for radioisotope incubations using ${}^{35}{S_{{H_{2}SO}_{4}^{2-}}}$ (Perkin Elmer) and ${}_{\text{ }}^{\text{34}}{\text{S}_{\text{H}_{\text{2}}\text{S}}}$ (synthesized as described above from ^34^S – Sigma Aldrich). 50 mL bottles of complete comproportionation medium were spiked with 1µCi ${}_{\text{ }}^{\text{35}}{\text{S}_{\text{SO}_{\text{4}}^{\text{2-}}}}$ and ${}_{\text{ }}^{\text{34}}{\text{S}_{\text{H}_{\text{2}}\text{S}}}$ (final concentration 1 mM, 50 at. %). After 24 hours of incubation at 15 mL, a 25 mL sample was taken for sulfur pool separation. 5 mL of medium was filtered through a 0.1 µm, 25 mm Supor filter to capture cells. The filters were then added to a scintillation vial and covered with 4.5 mL Ultima Gold scintillation cocktail (Perkin Elmer). Sulfate, sulfide, and S^0^ pools were separated. 50 mL of 5M NaOH was added to a 2 mL microcentrifuge tube in triplicate. With a syringe, 0.8 mL of the culture medium and 0.8 mL of 20% ZnCl_2_ were added to each of the 2 mL tubes to precipitate ZnS. The tubes were vortexed and centrifuged at 10,000 rpm for 10 minutes. The supernatant was removed and transferred to new tubes. 400 µL of 1M BaCl_2_ was added to the supernatant to precipitate BaSO_4_. The tubes were vortexed and centrifuged at 10,000 rpm for 10 minutes. The supernatant was removed and transferred to a scintillation vial. 2 mL of tetrachloroethylene was added to extract S^0^ (2) and the scintillation vials were shaken overnight. The following day, the organic layer was removed from the scintillation vial and transferred to a new vial. Both the aqueous and organic layers were covered with 4.5 mL scintillation cocktail. The precipitated ZnS and BaSO_4_ were resuspended in 1 mL diH_2_O and split into two scintillation vials. 4.5 mL Ultima Gold was added to one vial and the other was frozen for ^34^S measurements. Scintillation counting was performed on a Beckman Coulter LS 6000 Scintillation System.

*Transcriptomics*

Transcriptomics can be used to identify genes that are upregulated during comproportionation conditions. Cultures should be grown under comproportionation conditions and an alternative growth condition. For *Acidithiobacillus thiooxidans*, an alternative growth condition could be aerobic sulfide oxidation. Cells should be preserved with RNAlater (Thermo Fisher) and pelleted or collected on a sterile, 25 mm, 0.1 µm Supor filter. Total RNA can be extracted using the RNeasy Kit for RNA purification (Qiagen) and sequenced. Transcriptomic bioinformatic analysis can be performed as described by Novak and colleagues (5). Briefly, raw FASTQ files should be quality checked and aligned to existing genomes, and differential gene expression should be performed with DESeq2 (6) to compare the transcripts from the comproportionation condition and the alternative growth condition.

*Reduction potential calculations*

Reduction potentials for sulfur transformations were calculated following Thauer et al. 1977 (7)(7). Briefly, reactions were balanced with H_2_ as the reductant and values of ∆*G_r_*^0^ were calculated at 25ºC and 15ºC as described above. ∆*G_r_* was calculated with the activity of H^+^ set to 10^-7^ and all other activities set to 1. Eº was calculated from ∆G*_r_* using the Nernst equation, and ∆Eº was calculated by subtracting Eº_H2_ from Eº.


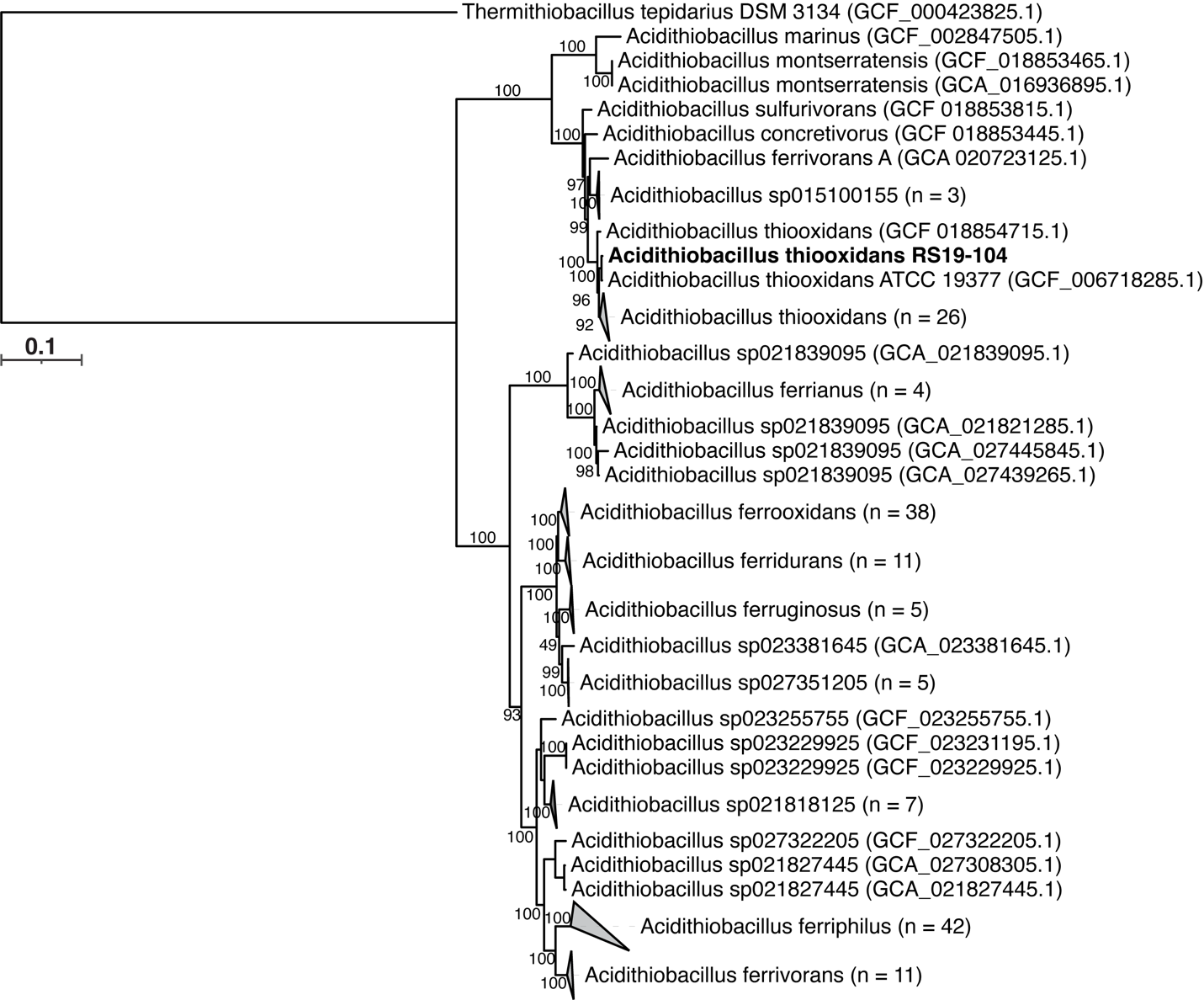


***Supplementary Figure 1:*** Maximum likelihood phylogenomic tree of 172 conserved Gammaproteobacterial single-copy marker genes from strain RS19-104 (bold) and representative genomes of members of the genus *Acidithiobacillus*. Bootstrap values (%) are based on 1000 replicates. NCBI accession numbers are shown in parentheses. Bar represents 0.1 substitutions per nucleotide position

***Supplementary Table 1:*** Gibbs energy yields, ∆*G_r_*, of potential catabolic reactions involving sulfate and ammonium under the chemical conditions of the custom comproportionation medium. These reactions are endergonic under medium conditions, making sulfide the only plausible reductant for sulfate in this system.

| **Reaction** | $\Delta G_{r}^{0}$ **(kJ mol^-1^)** | $\Delta G_{r}$ **(kJ mol^-1^)** |
| --- | --- | --- |
| $\text{4NH}_{\text{4}}^{\text{+}}\text{ + }\text{3SO}_{\text{4}}^{\text{2-}}\text{⇌}\text{ }\text{4NO}_{\text{2}}^{\text{-}}\text{ + }\text{3H}_{\text{2}}\text{S + 4}\text{H}_{\text{2}}\text{O}\text{ }\text{+ }\text{2H}^{\text{+}}$ | +1396 | +1414 |
| $\text{NH}_{\text{4}}^{\text{+}}\text{ + }\text{SO}_{\text{4}}^{\text{2-}}\text{ }\text{⇌}\text{ }\text{NO}_{\text{2}}^{\text{-}}\text{ + }\text{S}^{\text{0}}\text{ }\text{+}\text{ }\text{2}\text{H}_{\text{2}}\text{O}$ | +319 | +302 |
| $\text{NH}_{\text{4}}^{\text{+}}\text{ + }\text{SO}_{\text{4}}^{\text{2-}}\text{ }\text{⇌}\text{ }\text{NO}_{\text{3}}^{\text{-}}\text{ + }\text{H}_{\text{2}}\text{S}\text{ }\text{+}\text{ }\text{H}_{\text{2}}\text{O}$ | +450 | +418 |
| $\text{3NH}_{\text{4}}^{\text{+}}\text{ + }\text{4SO}_{\text{4}}^{\text{2-}}\text{ +2}\text{H}^{\text{+}}\text{ }\text{⇌}\text{ }\text{3NO}_{\text{3}}^{\text{-}}\text{ + }\text{4S}^{\text{0}}\text{+ 7}\text{H}_{\text{2}}\text{O}$ | +1229 | +1204 |
| $\text{8NH}_{\text{4}}^{\text{+}}\text{ + }\text{3SO}_{\text{4}}^{\text{2-}}\text{⇌}\text{ }\text{4N}_{\text{2}}\text{ + }\text{3H}_{\text{2}}\text{S + }\text{2H}^{\text{+}}\text{+ 12}\text{H}_{\text{2}}\text{O}$ | +18 | +75 |
| $\text{2NH}_{\text{4}}^{\text{+}}\text{ + }\text{SO}_{\text{4}}^{\text{2-}}\text{ }\text{⇌}\text{ }\text{N}_{\text{2}}\text{ + }\text{S}^{\text{0}}\text{+ 4}\text{H}_{\text{2}}\text{O}$ | -26 | +28 |

***Supplementary Table 2:*** Reduction potential of redox couples potentially involved in intracellular sulfur transformations.

| **Redox couple** | **Eº (mV) at 25ºC, pH 7** | **Eº (mV) at 15ºC, pH 6.5** |
| --- | --- | --- |
| SO_4_^2-^/HSO_3_^-^ | -515 | -451 |
| S^0^/H_2_S | -270 | -224 |
| SO_4_^2-^/S_4_O_6_^2-^ | -270 | -209 |
| SO_4_^2-^/S_2_O_3_^2-^ | -233 | -178 |
| SO_4_^2-^/S^0^ | -199 | -141 |
| S_2_O_3_^2-^/S^0^ | -132 | -66.2 |
| SO_3_^2-^/S^0^ | -41.3 | -16.0 |
| S_4_O_6_^2-^/S_2_O_3_^2-^ | 24.0 | 36.7 |
| SO_3_^2-^/S_2_O_3_^2-^ | 54.9 | 117 |
| SO_3_^2-^/S_4_O_6_^2-^ | 65.3 | 144 |

***Supplementary Tables 3-9 can be found in the excel sheet “SuppTables3-9”***

***Supplementary Table 3*:** Protein annotations from EggNOG-mapper2, MetaSanity, and Anvi'o. Column 1 lists the locus tags for each protein.

***Supplementary Table 4*:** Annotation output "HMMHitNum" from METABOLIC annotation software.

***Supplementary Table 5*:** Annotation output "FunctionHit" from METABOLIC annotation software.

***Supplementary Table 6*:** Annotation output "KEGGModuleHit" from METABOLIC annotation software.

***Supplementary Table 7*:** Annotation output "KEGGModuleStepHit" from METABOLIC annotation software.

***Supplementary Table 8*:** Annotation output "dbCAN2Hit" from METABOLIC annotation software.

***Supplementary Table 9*:** Annotation output "MEROPSHit" from METABOLIC annotation software

***Supplemental References***

1. J. D. Cline, Spectrophotometric Determination of Hydrogen Sulfide in Natural Waters. *Limnology and Oceanography* **14**, 454–458 (1969).

2. M. M. McGuire, R. J. Hamers, Extraction and quantitative analysis of elemental sulfur from sulfide mineral surfaces by high-performance liquid chromatography. *Environmental Science and Technology* **34**, 4651–4655 (2000).

3. P. Carini, A. E. White, E. O. Campbell, S. J. Giovannoni, Methane production by phosphate-starved SAR11 chemoheterotrophic marine bacteria. *Nat Commun* **5**, 4346 (2014).

4. D. Camacho, *et al.*, New Insights Into Acidithiobacillus thiooxidans Sulfur Metabolism Through Coupled Gene Expression, Solution Chemistry, Microscopy, and Spectroscopy Analyses. *Frontiers in Microbiology* **11**, 411 (2020).

5. J. K. Novak, P. G. Kennedy, J. G. Gardner, Transcriptomic analyses of bacterial growth on fungal necromass reveal different microbial community niches during degradation. *Appl Environ Microbiol* **90**, e01062-24 (2024).

6. M. I. Love, W. Huber, S. Anders, Moderated estimation of fold change and dispersion for RNA-seq data with DESeq2. *Genome Biol* **15**, 550 (2014).

7. R. K. Thauer, K. Jungermann, K. Decker, Energy conservation in chemotrophic anaerobic bacteria. *Bacteriological Reviews* **41**, 100–180 (1977).
