# Supplementary figures and images for: Isolation of a putative sulfur comproportionating microorganism
Source: Sci Rep. 2025 May 23;15:17999. doi: 10.1038/s41598-025-01009-y (PMC12102312; doi:10.1038/s41598-025-01009-y)

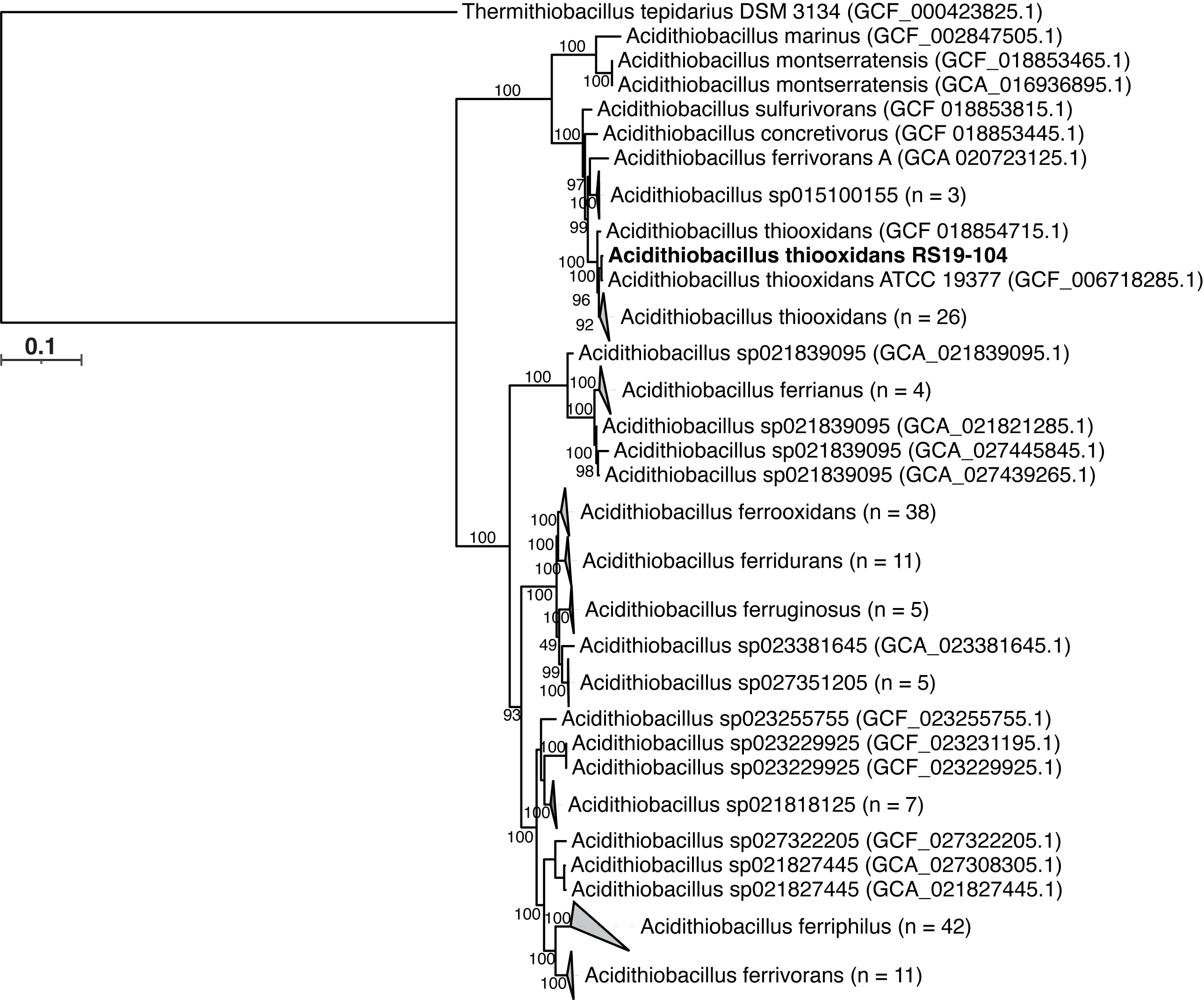

Supplement: Supplementary file 2 — Supplementary Material 2 [file 41598_2025_1009_MOESM2_ESM.tif]
